# Review of publication barriers in upper limb absence rehabilitation: A protocol for innovative literature macro-analyses

**DOI:** 10.1371/journal.pone.0360504

**Published:** 2026-10-23

**Authors:** Laurent Frossard, Beth Sheehan

**Affiliations:** 1 YourResearchProject, Brisbane, Australia; 2 Queensland University of Technology, Brisbane, Australia; 3 International Confederation of Amputee Associations (IC2A), Stuttgart, Germany; 4 Beth Sheehan Consultancy, Brisbane, Australia; 5 International Society of Wheelchair Professionals, Pittsburgh, United States of America; Roma Tre University: Universita degli Studi Roma Tre, ITALY

## Abstract

A preliminary review of the literature suggested that rehabilitation programs for upper limb absence (ULA) may be constrained by several publication barriers. Typically, clusters of publications concentrating on a particular topic may be analyzed utilizing bibliometric or scientometric approaches. Practically, these conventional methods might be inadequate to examine these barriers as the literature about ULA rehabilitation includes hundreds of publications. The purpose of this review protocol was to introduce new literature macro-analyses utilizing ratio-based metrics to characterize the alleged publication barriers in ULA rehabilitation. The objectives were to outline the steps for literature search strategies, reference selection and analyses of the barriers focusing on growth, expertise, location, apportion, strength, population and pertinence of the literature. The protocol will follow a 16-step process including 76 tasks. The searches, organization and extraction of references will be performed following the PRISMA guidelines. A 37-point Reference Acquisition Form recording data for macro-analyses will be completed for each reference. The macro-analyses will investigated if the literature: has increased noticeably in recent years (growth); is essentially generated by a limited number of authors (expertise); is mainly USA-centric (location); does unevenly represent populations from countries with different income levels (apportion); is mainly populated by studies with limited evidence (strength); is mainly populated by studies involving a limited population (population); does unevenly focus on care interventions and clinical outcomes (pertinence). The characterization of the barriers will be limited by the mining of the source data such as estimates of the global population and inconsistent metadata across sources. It will also be limited by the interpretation of ratio-based metrics that will be inevitably subjective due to the lack of comparative data. Hopefully, subsequent reviews will assist decision-makers in selecting strategies that enhance bespoke rehabilitation programs, facilitate prosthesis use, and improve quality of life for individuals with upper limb absence.

## Introduction

### Current understanding of global barriers

Healthcare and socioeconomic support available to individuals with upper limb absence (ULA) frequently fall short of adequately addressing their medical, social, and financial needs [1–4]. This leads to a significant rate of prosthetic abandonment and adversely impacts their quality of life [2,3,5–10].

A preliminary review of the literature on ULA rehabilitation suggested that difficulties in achieving satisfactory outcomes may be due, either partially or entirely, to the influence of prevailing seven publication barriers outlined in Table 1A.

**Table 1:** Summary of (A) alleged publication barriers in upper limb absence rehabilitation as well as (B) the aims, objectives, assessments and typical limitations of literature macro-analyses. NoR: Number of references. NoP: Number of participants, ULA: Population including individuals with upper limb absence, ABD: Population including able-bodied or asymptomatic participants, UNA: Population including both ULA and ABD cases, NoR/M: Number of references per million of individuals with ULA, Q1-Q4: Interquartile Range.

A-Barriers	B-Macro-analyses			
**Description**	**Aim**	**Objectives**	**Assessment**	**Limitations**
***The literature focusing on ULA rehabilitation…***	***The aim will be …***	***The objective will be to present…***	***The barrier will be evidenced by…***	***The analysis will be limited by…***
**A1-Growth** [11,20]				
Has increased noticeably in recent years	Describe the historical progression of the literature	The annual NoR extracted	The annual NoR between 1900 and 2024	Potential underestimation of the NoR; Considering a linear projection based on growth over the last five years
		The cumulative NoR extracted	The cumulative NoR from 1900 to 2024	
		The growth of the NoR extracted	Interquartile range of cumulated NoR up to 2024	
	Forecast the NoR based on current growth	The NoR projected over a 10-year horizon	The estimated of NoR by 2033	
	Determine the representativity of the subsequent macro-analyses	The NoR selected compared to the NoR extracted	The selection ratio: NoR extracted/ NoR selected	
**A2-Expertise** [11]				
Is essentially generated by a limited number of authors	Characterize the expertise pyramid and the critical mass of experts contributing to the literature	Number of authors at each expertise level (i.e., novice, specialist, and expert) according to their publication counts	The expertise ratios: novice/ expert authors and specialist/ expert authors	Overlooking position of authors within the byline; Overlooking the impact of the publications (e.g., citations); Lack of standards about the preferable shape of expertise pyramid, critical mass of experts and expertise ratios; Overlooking conflict of interest
**A3-Location** [11,16,21,22]				
Is mainly USA-centric	Identify the locations where the knowledge was generated	The NoR produced by countries and continents of origin	The continent ratio: NoR from North America/ NoR from Europe	Absence of map showing leading organizations (e.g., hotspots)
**A4-Apportion** [11,14,23,24]				
Does unevenly represent populations from countries with different income levels	Describe the differences between continents in the NoR relative to the regional population of individuals living with ULA	The NoR per million individuals with ULA in North America, Europe, Australia, South America, Asia and Africa	The apportion ratios for NoR/M in North America, Europe, Australia, South America, Asia and Africa	Potential inaccuracy of population data; No evidence that the NoR in a region equates to better understanding of the ULA rehabilitation in this region
**A5-Strength** [15,16,20,25]				
Is mainly populated by studies with limited evidence	Show the strength of the literature considering study designs and the associated levels of evidence	The NoR per level of development (basic and advanced), synthesis (primary and secondary), and evidence (low, high)	The development ratio: NoR with basic studies/ NoR with advanced studies	Educated choices to categorize study type (e.g., levels of evidence, development, synthesis); Biasing classification toward low level on evidence by including case control and cohort studies; No direct link between levels of development (e.g., conversion rate from primary to secondary studies); No direct association between levels of synthesis (e.g., inclusion rate of references per review); Overlooking methodological rigor and quality (e.g., blinding, sample size, randomization statistical power, risks of bias)
			The synthesis ratio: NoR with primary studies/ NoR with secondary studies	
			The evidence ratio: NoR with low level/ NoR with high level	
**A6-Population** [15–17,20,26–29]				
Is mainly populated by studies involving a limited population	Determine the sample size of primary studies	The NoP per study for each population	The median NoP per study for ULA, ABD and UNA population	No stratified analyses by studies setting, gender, racial ethnicity, cause and level of amputation; No differentiation between survey-based studies with larger sample sizes due to easier recruitment methods (e.g., self-report, phone interviews) and studies assessing physical tasks facing challenges in recruiting participants (e.g., motion capture, movement analysis); Overlooking whether the ABD population was considered independently or as a control or comparator group (e.g., selection and randomization process)
	Show the distribution of references per study populations	The NoR involving ULA, ABD and UNA populations	The case ratios: NoR with ULA/ NoR with ABD, NoR with ULA/ NoR with UNA	
Is including an important relative number of ABD participants	Show the relative NoP with ULA relative to ABD in UNA population	The NoP with ULA relative to number of ABD participants studied involving UNA population	The sample ratio: NoP with ABD/ NoP with ULA in studies with UNA population	
**A7-Pertinence**				
Does unevenly focus on care interventions and clinical outcomes	Show the distribution of references focusing on care interventions and/or clinical outcomes	The NoR for each of 7 levels of evidence focusing on 3 topics focusing care interventions (e.g., device, control, emerging solutions) and/or 4 topics focusing clinical outcomes (e.g., neuromusculoskeletal pain, fitting satisfaction, ambidexterity level, functional ability)	Heatmap of NoR per level of evidence focusing care interventions and clinical outcomes	Detection of keywords without contextual analyses; Overlooking methodological rigor and quality (e.g., blinding, sample size, randomization statistical power, risks of bias)

These barriers commonly highlighted in the discussion sections of publications, may appear anecdotal but they are undoubtedly insightful. In all cases, these alleged barriers provided a strong foundation for further quantitative validations.

### Needs for literature macro-analyses

Practically, a fraction of the literature can be systematically retrieved from databases (e.g., Google Scholar, PubMed, Web of Science, Scopus). Clusters of publications can be analyzed using several methods such as bibliometric studies, citation mapping, keyword tracking, and trend visualization (e.g., Google Trends, VOSviewer, CiteSpace, CitNetExplorer Bibliometrix) [11].

Furthermore, limited series of publications can be systematically evaluated using various established quantitative bibliometrics or scientometrics (e.g., annual number of publications, keyword frequency, citation counts, H-index, impact factor) [11,12].

Primarily designed to facilitate systematic quantitative literature reviews bridging the gap between traditional narrative review methods and meta-analyses, these metrics are effective to appraise clusters of publications that concentrate on a particular topic (e.g., myoelectric hand, cost analyses, virtual reality, neurorehabilitation, mirror therapy) [13–19]. The quality of systematic reviews can be maintained by adhering to the Preferred Reporting Items for Systematic Reviews and Meta-Analyses (PRISMA) guidelines and checklist.

Unfortunately, these traditional systematic review and meta-analysis approaches are impractical for evaluating an entire field such as the ULA rehabilitation, which encompasses hundreds of publications. Challenges such as data dimensionality, standardization requirements, and visualization overload make these approaches difficult to implement [11]. Consequently, the ability of these metrics to characterize the barriers outlined in Table 1A is limited.

Therefore, there is a need for new large-scale metrics that can comprehensively assess the body of literature in the field of ULA rehabilitation ensuring that references are searched, organized, and extracted in line with PRISMA guidelines.

Such metrics should be able to quantify the purported barriers, determine the current status of literature development, and potentially guide decisions that foster global research advancements in ULA rehabilitation.

### Purpose

Expanding upon conventional systematic reviews, this review protocol introduces innovative literature macro-analyses utilizing purposely designed ratio-based metrics to characterize the alleged barriers in ULA rehabilitation.

The objective is to outline the 16-step process including 76 tasks required to conduct the literature search strategies, reference selection and, more importantly, analyses of the publication barriers examining growth, expertise, location, apportion, strength, population and pertinence in the literature.

## Materials and methods

The literature macro-analyses of the barriers will be conducted following a comprehensive 16-step process presented in Fig 1, including 76 tasks detailed in Table 2. Steps 1–8 will focus on the searches, organization and extraction of references in accordance with the PRISMA guidelines.

**Table 2:** Overview of 16-step process and description of 76 tasks used to (A) conduct searches, (B) select references and (C) perform the literature macro-analyses using EndNote 20 library (Clarivate Analysis LLC, United Kingdom) and Excel spreadsheet (Microsoft, Microsoft 365, version 2507, USA). ULA: Population including individuals with upper limb absence, PICOS: Population, intervention, comparator, outcome and design elements of studies, CINAHL: Cumulative index of nursing and allied health literature, RAF: Reference acquisition form, CSV: Comma separated values, IQR: Interquartile range, ISO: International organization for standardization, NoR/M: Number of references per million of ULA per million of population, PRISMA: Preferred reporting items for systematic reviews and meta-analyses, PROSPERO: International prospective register of systematic reviews, L: Levels of evidence, ABD: Population including able-bodied or asymptomatic participants, UNA: Population including both ULA and ABD cases.

Task	Task description	Reference
**A. Conduct searches**		
**Step 1. Conduct preliminary work**		
1	Import authors' personal reference about ULA into new EndNote library	
2	Extract and adapt keywords and syntax from examples provided in the literature	[3, 20]
**Step 2. Perform generic searches**		
3	Identify 91 keywords around the PICOS process including 11 keywords related to population, 33 keywords related to interventions (e.g., attachment, terminal device, body powered, externally powered, hybrid prostheses), 37 keywords related to broad outcomes for rehabilitation (e.g., pain, fitting, handedness, function), and 10 keywords related to study design	
4	Perform generic searches including input keyword permutations into electronic databases (e.g., PubMed, Web of Science, Scopus, Central Cochrane Register of Controlled Trials, CINAHL, Google Scholar) and clinical care guideline-specific websites (e.g., Australian National Health and Medical Research Council, International Network, National Guideline Clearinghouse, National Institute for Health and Clinical Excellence, Scottish Intercollegiate Guidelines Network, Turning Research Intro Practice database, World Health Organization website)	
**Step 3. Perform targeted searches**		
5	Identify 344 keywords focusing on: residuum health (e.g., neuromusculoskeletal pain, fitting satisfaction, ambidexterity level, functional ability), intervention (e.g., device, control), emerging solutions (e.g., surgical treatments) and health economics (e.g., cost-utility analysis)	
6	Perform targeted searches into electronic databases (e.g., PubMed, Web of Science, Scopus, Central Cochrane Register of Controlled Trials, CINAHL, Google Scholar) and clinical care guideline-specific websites (e.g., Australian National Health and Medical Research Council, International Network, National Guideline Clearinghouse, National Institute for Health and Clinical Excellence, Scottish Intercollegiate Guidelines Network, Turning Research Intro Practice database, World Health Organization website)	
**B. Select references**		
**Step 4. Organize references**		
7	Import references found in the initial searches into EndNote library	
8	Remove duplicates references	
9	Organize references using a mix of group sets and smart groups according to specific design and topics related to PICOS process	
**Step 5. Screen references**		
10	Assess each reference considering titles and abstracts independently by two reviewers	
11	Consult with a third reviewer in cases of disagreement regarding the eligibility and assessments	
12	Conduct first screening of extracted references published between 1900 and 2024 in EndNote library	
**Step 6. Apply exclusion criteria**		
13	Exclude references published in a language other than English	
14	Exclude references focusing on animals, lower limb and rubber hand illusion, spinal cord injuries and tetraplegia	
**Step 7. Apply inclusion criteria**		
15	Include reviews and clinical guidelines as well as any other types of observational and qualitative studies	
16	Include references with participants of any age, gender, background and upper limb absence conditions (e.g., congenital, acquired) if they were related to ULA rehabilitation	
**Step 8. Export extracted and selected references**		
17	Export extracted references published between 1900 and 2024 into a CSV file	
18	Export selected references published between 2018 and 2024 into a CSV file	
**C. Perform literature macro-analyses**		
**Step 9. Complete Reference Acquisition Form (RAF)**		
19	Import references into a purposely designed spreadsheet	
20	Populate automatically RAF with whole citation and fields imported for each reference (i.e., title and abstract, link, authors, affiliations, cities of origin, countries of origin, year of publication, dataset ID)	
21	Populate manually the RAF with the information tabulated by the operators (i.e., case, sample size, study design, comment) for each reference	
22	Export RAF information for references selected into CSV file	
**Step 10. Complete growth analysis**		
23	Import references extracted from CSV file onto purposely designed spreadsheet	
24	Extract the year of publication for each reference	
25	Sum up the annual and cumulative NoR	
26	Report the growth of the literature per interquartile range (IQR) where the total NoR in 2024 is 100%	
27	Report the relative representation of the NoR selected based on the annual percentage of the extracted references	
28	Forecast the NoR by 2033, extrapolating from linear projection based on growth over the last 5 years	
29	Calculate the selection ratio rounded to the nearest integer between the total NoR extracted and selected	
**Step 11. Complete expertise analysis**		
30	Import RAF information for references selected published between 2018 and 2024 from CSV file onto purposely designed spreadsheet	
31	Extract the byline of authors from the RAF for each reference and identify individual authors by their last name and up to two initials for the first and middle names, depending on the sources	
32	Calculate the frequency of authorship corresponding to the number of times an author was found amongst all references selected	
33	Categorize authors as novice, specialist and expert when they authored with up to 5, between 6 and 10, and more than 11 references, respectively	
34	Calculate the pyramid of expertise using the expertise ratios rounded to the nearest integer between number of novices and specialists per expert authors	
**Step 12. Complete location analysis**		
35	Import RAF information for references selected between 2018 and 2024 from CSV file onto purposely designed spreadsheet	
36	Extract semi-automatically all affiliations including the countries of origin from the RAF for each reference	
37	Differentiate affiliation despite random mishmashes in names of organization (e.g., departments, universities, industries, hospitals, medical centres, governmental organizations) and cities or countries of origin	
38	Extract countries and continents following the ISO 3166 country code	
39	Conduct an affiliation-based analysis where countries were counted for each affiliation	
40	Conduct a country-focused analysis where a country was counted only once even if it appeared in multiple affiliations	
41	Calculate the continent ratios rounded to the nearest integer between NoR originating from North America and Europe	
**Step 13. Complete apportion analysis**		
42	Import RAF information for references selected between 2018 and 2024 from CSV file onto purposely designed spreadsheet	
43	Identify the number of individuals living with ULA reported in terms of prevalence or incidence considering based estimations of incidence of ULA varies depending on the focus of the publication (e.g., aetiology, location)	[30–32]
44	Aggregate relative population expressed in millions of individuals leaving with ULA from the prevalence data for non-fatal trauma ULA	[31, 32]
45	Consider estimation from McDonald et al (2021) that 21.7 Million people live with traumatic non-fatal upper limb amputation (i.e., falls, road injuries, other transportation injuries, mechanical forces) in 2017, with 66% of this population located in East Central Asia, Europe, and South Asia	[31]
46	Adjust initial North American dataset considering Caruso and Harrington (2024) estimating that nearly 2.3 million are people living ULA in the United States	[32]
47	Consider NoR produced in each continent extracted from the country-focused location analysis	
48	Calculate and compare apportion ratio between the NoR per million of ULA (NoR/M) for each continent, so that high apportion ratio indicates a substantial scientific output relative to the population in a continent	
**Step 14. Complete strength analysis**		
49	Import RAF information for references selected between 2018 and 2024 from CSV file onto purposely designed spreadsheet	
50	Investigate literature focusing on criterium for a particular study type showing there are inconsistently of definitions between fields and reviews as well as unreliability when the author's self-report their study design	
51	Consider the data from Geil (2016) showing that studies published in Journal of Orthotics and Prosthetics in 2015 involving human participants had sample size ranging from 3 to 41, with an average number of 14	[33]
52	Consider the data from Beaudette and Fiedler (2018) showing that 32 studies focusing on prosthetic knee componentry had on average around 20 participants and rarely more than 50 participants	[34]
53	Consider that studies involving ten or less participants will be conservative categorised as "case series"	
54	Create primary studies categories including four types of observational studies involving participants (i.e., case series, cohort study, case control, randomized trial)	
55	Create secondary studies categories including three types of reviews studies (i.e., expert opinion, narrative, literature, systematic reviews) depending on consideration and outline of searches, compliance with PRISMA guideline and/or registration in PROSPERO	
56	Allocate a level of evidence (L) to each type of study design according to typical pyramid of evidence	[20]
57	Identify one of the 10 types of study design for each reference semi-automatically based on information provided in the title and abstract against set criteria	
58	Duplicate entries for references including mix-methods so that these references appear for each type in the spreadsheet (e.g., literature review and cohort study)	
59	Calculate evidence ratio rounded to the nearest integer corresponding to the NoR with low level below L5 (i.e., technical note, expert opinion, case series, narrative review, case control, cohort Study) per high level above L4 (i.e., literature review, randomized trial, systematic review, practice guideline)	
60	Calculate the development ratio rounded to the nearest integer corresponding to the number of basic (i.e., technical note, case series) per advanced (i.e., cohort study, case control, randomized trial) studies	
61	Calculate the synthesis ratio rounded to the nearest integer corresponding to the number of primary studies per secondary study	
**Step 15. Complete population analysis**		
62	Import RAF information for references selected between 2018 and 2024 from CSV file onto purposely designed spreadsheet	
63	Identify case and sample size manually for each reference selected considering information provided in the title and abstract and eventually the manuscript when needed	
64	Extract the number of participants for each three cases involving upper limb absence (ULA), able-bodied or asymptomatic participants (ABD) or both ULA and ABD (UNA)	
65	Conduct preliminary inspection of the dataset to identify possible outliers references presenting disproportionated sample size	[35, 36]
66	Apply the modified IQR method to confirm check for outliers (e.g., cohort sizes above the upper bound [Third Quartile or Q3+40xIQR])	
67	Remove outliers from the analyses to reduce biases due to uniquely well-powered studies based on queries of large databases, if needed	[35, 36]
68	Represent the number of participants per condition using the median and IRQ or mean and standard deviation depending on non-normal or normal distribution of the population	
69	Calculate the case ratios rounded to the nearest integer between NoR involving participants with ULA per NoR involving ABD and UNA populations	
70	Calculate the sample ratio rounded to the nearest integer between the number of ABD participants per NoP with ULA in studies with UNA population	
**Step 16. Complete pertinence analysis**		
71	Import RAF information for references selected between 2018 and 2024 from CSV file onto purposely designed spreadsheet	
72	Search title and abstract for keywords related to three care interventions focusing on device, control, emerging solutions (e.g., osseointegration, regenerative peripheral nerve interfaces, targeted muscle reinnervation)	
73	Search title and abstract for keywords related to four clinical outcomes including neuromusculoskeletal pain, fitting satisfaction, ambidexterity level, functional ability	
74	Paire references focusing on any of interventions and outcomes based on occurrences of keywords.	
75	Calculate an evidence score combining the number of publications and level of evidence according to study design calculated for each association	
76	Represent the evidence score for each association between topics related to interventions and outcomes as an heatmap where the spectrum from light to dark is proportional to the highest and lowest scores, respectively.	

**Fig 1:**
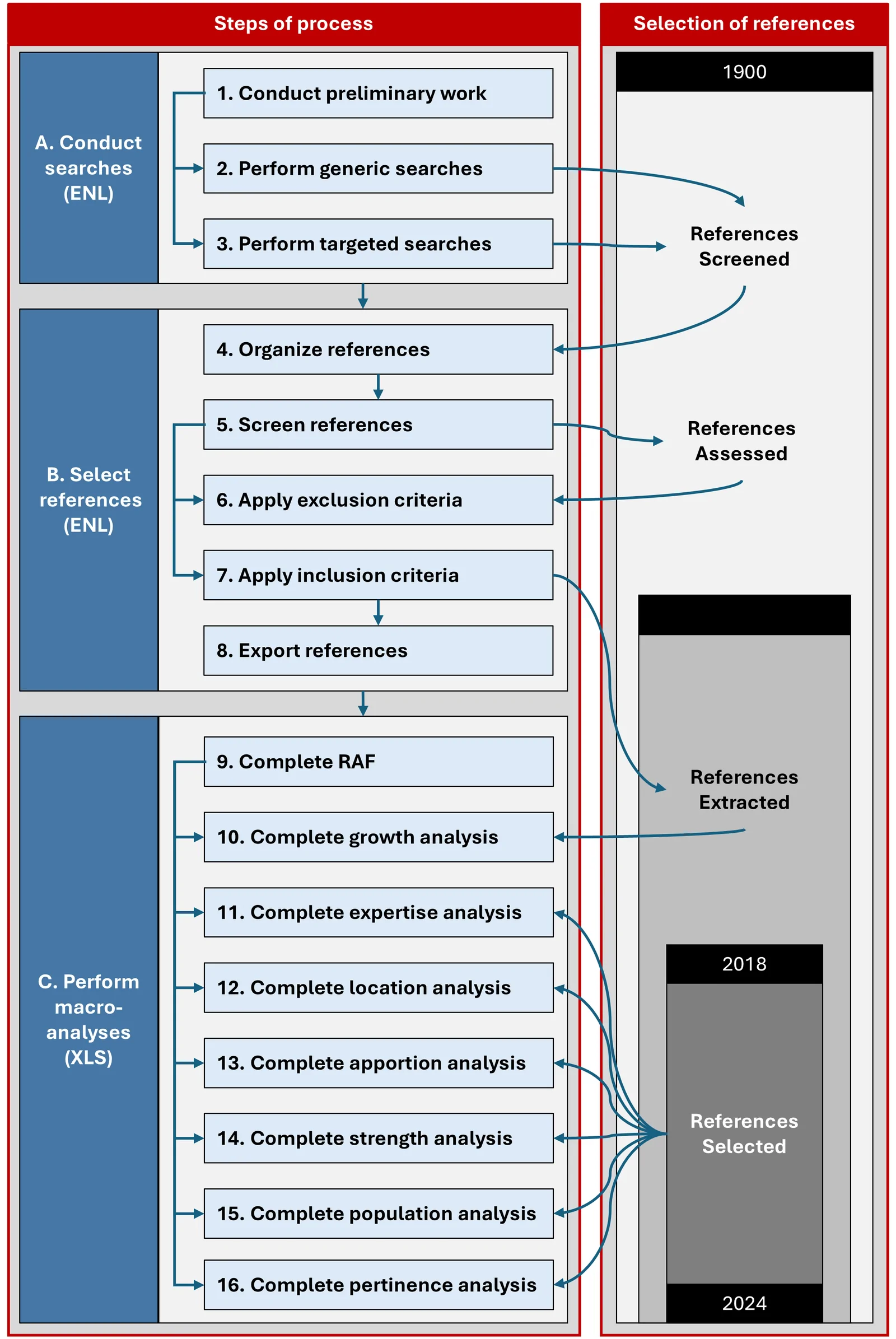
Dataflow for the 16-step process to (A) conduct searches, (B) select references and (C) perform the literature macro-analyses alongside the timespan for the references screened, assessed, extracted and selected (2018-2024). ENL: EndNote 20 library (Clarivate Analysis LLC, United Kingdom), XLS: Excel spreadsheet (Microsoft, Microsoft 365, version 2507, USA), RAF: Reference Acquisition Form.

### Searches strategies (Table 2, Steps 1–3)

The authors’ personal libraries will be complemented by computerized literature searches organized around the permutation of 344 keywords provided in S1 Table.

Generic searches will involve broad keywords associated with the population, intervention, comparator, outcome and design (PICOS) elements of studies.

Targeted searches will involve specific keywords related to residuum health, clinical care intervention, emerging solutions and health economics.

Both generic and targeted search will be conducted in common electronic databases (e.g., PubMed, Web of Science, Scopus, Central Cochrane Register of Controlled Trials, CINAHL, Google Scholar) and clinical care guideline-specific websites.

Examples of searches including focus, subtopic, source and syntax are presented in S2 Table.

### References selection (Table 2, Steps 4–8)

Reviewers will purposely consider a broad range of scientific publications from the searches, including clinical guidelines and literature reviews as well as any other types of observational and qualitative studies.

References will be screened and assessed by two reviewers independently considering titles and abstracts. A third reviewer will be consulted if there is disagreement about eligibility or assessments.

Reviewers will exclude references published in a language other than English as well as those focusing only on animals, lower limb and rubber hand illusion, spinal cord injuries and tetraplegia.

References including participants of any age, gender, ethnicity, background and upper limb absence conditions (e.g., congenital, acquired) will be included if they pertain to ULA rehabilitation.

### Literature macro-analyses (Table 2, Steps 9–16)

References will be imported into a purposely designed 37-point Reference Acquisition Form (RAF) presented in Table 3. This RAF will display citation details (e.g., title and abstract, link, authors, affiliations, cities of origin, countries of origin, year of publication, dataset ID). Reviewers will also use the RAF to tabulate information required for the macro-analyses of barriers (e.g., case, sample size, study design, comment).

**Table 3:** Reference Acquisition Form (RAF) including 37 datapoints to assess the citation imported from EndNote library and information tabulated by the reviewers. X: datapoint, NoP: number of participants, ULA: Population including individuals with upper limb absence, ABD: Population including able-bodied or asymptomatic participants, UNA: Population including both ULA and ABD cases.

Reference Acquisition Form						
Full reference			Completion rate			
Reference data and dataset						
Section	Variables	Unit	Information			
1	Reference					
1.1	Data entry					
1.1.1	Publication ID	(#)	x			
1.1.2	EndNote Nb	(txt)	x			
1.1.3	Reviewer	(txt)	x			
1.1.4	Date	(dd/mm/yyyy)	x			
1.2	Publication					
1.2.1	Title and abstract	(txt)	x			
1.2.2	Link	(txt)	x			
1.2.3	Authors	(txt)	x			
1.2.4	Affiliations	(txt)	x			
1.2.5	Cities of origin	(txt)	x			
1.2.6	Countries of origin	(txt)	x			
1.2.7	Year of publication	(yyyy)	x			
1.2.8	Dataset ID	(#)	x			
1.3	Selection					
1.3.1	Language	(txt)	x			
1.3.2	Aims of study	(txt)	x			
1.3.3	Inclusion in review	(0/1)	x			
1.3.4	Reason for exclusion	(txt)	x			
2	Population					
2.1	Type of population					
			**ULA**	**ABD**	**UNA**	**Total**
2.1.1	Case	(0/1)	x	x	x	x
2.1.2	Sample	(NoP)	x	x	x	x
3	Design					
3.1	Type of design					
3.1.1	Unclear	(0/1)	x			
3.1.2	Technical note	(0/1)	x			
3.1.3	Expert opinion	(0/1)	x			
3.1.4	Case series	(0/1)	x			
3.1.5	Narrative review	(0/1)	x			
3.1.6	Case control	(0/1)	x			
3.1.7	Cohort Study	(0/1)	x			
3.1.8	Literature review	(0/1)	x			
3.1.9	Randomized trial	(0/1)	x			
3.1.10	Systematic review	(0/1)	x			
3.1.11	Practice guideline	(0/1)	x			
4	Comment					
4.1	Reviewer 1					
4.1.1	Comment	(txt)	x			
4.2	Reviewer 2					
4.2.1	Comment	(txt)	x			

Based on the study recently published by Liu et al (2024) about global trends and current status of literature about limb amputation, we anticipate that up to two thousands references will be considered [11]. Therefore, it will be unfeasible to consider all these references for the in-depth macro-analyses, other than the growth analysis.

Consequently, only references published between 2018 and 2024 will be considered for the other macro-analyses. The lower year limit of 2018 corresponds with the end of the literature searches conducted by Heyms et al (2021) [25]. This study was the latest systematic review considering standard of care and clinical practice guidelines for management of ULA provided by certified peak bodies.

In this review, the term “literature” will refer to references extracted published since 1900 including the references selected published between 2018 and 2024 (Fig 1).

Information from the RAFs will be imported into purposely designed spreadsheets to conduct each macro-analysis.

The aims and methods summarized on Table 1 and Table 2C are described in detail below for each macro-analysis.

#### Growth analysis (Table 2, Step 10).

As presented Table 1A1, the growth analysis will investigate if the literature has increased noticeably in recent years. This analysis will provide a comprehensive overview of the long-term historical progression of the literature by examining annual, cumulative, and interquartile range (IQR) of number of references (NoR) extracted from 1900 through 2024. The analysis will also forecast the NoR over a ten-year period. Finally, this analysis will assess the representativeness of the subsequent macro-analyses using a selection ratio defined as the NoR extracted per reference selected.

As detailed in Table 2C (Tasks 23–29), the year of publication will be extracted from the RAF for each reference and sum up to provide the annual and cumulative NoR. The growth of the literature will be reported per IQR where the total NoR in 2024 will be 100%. The NoR projected by 2034 will be extrapolated from linear projection based on growth over the last 5 years. The relative representation of the NoR selected will be based on the annual percentage of the references extracted and the selection ratio.

#### Expertise analysis (Table 2, Step 11).

As presented Table 1A2, the expertise analysis will investigate if the literature is essentially generated by a limited number of authors. The analysis will characterize the expertise pyramid and the critical mass of experts contributing to the literature. These aims will be achieved by quantifying the number of authors at each expertise level according to their publication counts (i.e., novice, specialist, and expert). The distribution of authors across levels of expertise will be evidenced using expertise ratios defined as the number of novices and specialists per expert author.

As detailed in Table 2C (Tasks 30–34), the byline of authors will be extracted from the RAF. Authors will be identified individually by their last name and up to two initials for the first and middle names, depending on the sources. The frequency of authorship will correspond to the number of times an author is identified in a byline. Authors will be categorized as novice, specialist and expert if they are named in 5 or less, between 6 and 10, and more than 11 references, respectively.

#### Location analysis (Table 2, Step 12).

As presented Table 1A3, the location analysis will investigate if the literature is mainly USA-centric. The analysis will identify the locations where the knowledge was generated. This aim will be achieved by extracting the NoR produced by countries and continents of origin. The distribution of NoR across continents will be evidenced using continent ratio defined as the NoR from North America per NoR from Europe.

As detailed in Table 2C (Tasks 35–41), all affiliations will be extracted semi-automatically from the RAF anticipating that they will be inconstancies in the names of organizations and/or cities and countries of origin between references. In all cases, countries and continents will be extracted following the ISO 3166 country code. The challenge of inventorying countries of origin in references with multiple affiliations will be overcome by conducting an affiliation-based analysis where countries will be counted for each affiliation, and country-focused analysis where a country will be counted only once even if it appears in multiple affiliations.

#### Apportion analysis (Table 2, Step 13).

As presented Table 1A4, the apportion analysis will investigate if the literature does unevenly represent populations from countries with different income levels. The analysis will describe the differences between continents in the crude NoR relative to the regional population of individuals living with ULA. This aim will be achieved by comparing continent ratios defined as the NoR per million of ULA in North America, Europe, Australia, South America, Asia and Africa.

As detailed in Table 2C (Tasks 42–48), the population of individuals with ULA for North America, Europe, Australia, South America, Asia and Africa will be extracted from Table 4. We generated this table based on regional distribution of individuals living with ULA due to non-fatal trauma in 2017 (i.e., falls, road injuries, other transportation injuries, mechanical forces) extracted from a market landscape analysis [30] and adjusted using McDonald et al (2021) [31] and Caruso and Harrington (2024) [32] as detailed in Tasks 43, 44, and 45 in Table 2C, respectively. The NoR produced in each continent will be extracted from the country-focused location analysis described above.

**Table 4:** Total and regional distribution of individuals living with upper limb amputation due to non-fatal trauma in 2017 (i.e., falls, road injuries, other transportation injuries, mechanical forces) extracted from market landscape analysis [30] and adjusted using McDonald et al (2021) [31] and Caruso and Harrington (2024) [32]. % of ULA: Percentage of upper limb amputation in relation to total limb amputations.

Ranking	Region	Total	Upper limb		
		(Million)	(% of ULA)	(Million)	(% per region)
1	East Central Asia	17	38	6.46	30
2	Europe	14	29	4.06	19
3	South Asia	11	34	3.74	17
4	Africa	5	25	1.25	6
4	North America	5	41	2.05	9
4	Middle East	5	26	1.3	6
7	Central South America	4	40	1.6	7
8	Southeast Asia	3	30	0.9	4
9	Australia	1	34	0.34	2
	**Total**	**65**		**21.7**	**100**

#### Strength analysis (Table 2, Step 14).

As presented Table 1A5, the strength analysis will investigate if the literature is mainly populated by studies with limited evidence. This analysis will show the distribution of the NoR per levels of evidence, development and synthesis according to study designs. This aim will be achieved using (A) evidence ratio defined as the NoR with low per high level of evidence, (C) development ratio defined as the number of basic per advanced studies and (C) a synthesis ratio defined as the number of primary per secondary studies.

As detailed in Table 2C (Tasks 49–61), reviewers will allocate one of the 10 types of study design to each reference semi-automatically based on information provided in the title and abstract against criteria presented in Table 5. Arguably, we will categorized studies involving ten or less participants as “case series”. The threshold is deemed conservative to differentiate case series and cohort studies based on information provided in the literature. Geil (2016) indicated that studies published in Journal of Orthotics and Prosthetics in 2015 involving human participants had sample size ranging from 3 to 41, with an average number of 14 [33]. Beaudette and Fiedler (2018) highlighted that 32 studies focusing on prosthetic knee componentry had on average around 20 participants and rarely more than 50 participants [34]. The three types of review studies will be categorized depending on consideration and outline of searches, compliance with PRISMA guideline and/or registration in PROSPERO.

**Table 5:** Description of 10 study designs as well as level of evidence, development and synthesis. L: Level of evidence, ULA: Population including individuals with upper limb absence, ABD: Population including able-bodied or asymptomatic participants, PRISMA: Preferred Reporting Items for Systematic Reviews and Meta-Analyses, PROSPERO: International Prospective Register of Systematic Reviews.

Study design		Description	Evidence		Development	Synthesis
1	Technical note	Focus on practical element of intervention without human participant	L1	Low	Basic	Primary
2	Expert opinion	Present view on specific topic without formal literature review	L1	Low	–	Secondary
3	Case series	Involve a total of ten or less ULA and/or ABD participants	L2	Low	Basic	Primary
4	Narrative review	Review of literature without clear outline of searches	L2	Low	–	Secondary
5	Case control	Involve the comparison of at least two groups of ULA and/or ABD participants	L3	Low	Advanced	Primary
6	Cohort Study	Involve more than ten ABD and/or ULA participants	L4	Low	Advanced	Primary
7	Literature review	Review the literature with outline of searches but no clear outline of PRISMA guideline and/or no registration in PROSPERO	L5	High	–	Secondary
8	Randomized trial	Outline randomization of cohorts of ULA and/or ABD participants	L5	High	Advanced	Primary
9	Systematic review	Review the literature with clear outline of PRISMA guideline and/or registration in PROSPERO	L6	High	–	Secondary
10	Practice guideline	Present practice guidelines including list of recommendations	L7	High	–	Secondary

The level of evidence (L) will correspond to the quality of a study’s design assigned according to the standard pyramid hierarchy; as the methodological rigor advances, the level increases by one point for each successive type. Study design score below L5 (i.e., technical note, expert opinion, case series, narrative review, case control, cohort study) and above L4 (i.e., literature review, randomized trial, systematic review, practice guideline) will be associated with low and high level of evidence, respectively.

The level of development of study design will be categorized as either basic or advanced. Basic design will include studies focusing on practical elements of intervention involving less than 10 participants (i.e., technical note, case series). Advanced design will include studies involving one or more series or cohort of participants (i.e., cohort study, case control, randomized trial).

The level of synthesis of study design will be categorized as either primary or secondary. Primary design will include four types of observational studies involving participants (i.e., case series, cohort study, case control, randomized trial). Secondary design will include opinion or review studies aggregating raw data presented in primary studies (i.e., expert opinion, narrative, literature, systematic reviews).

#### Population analysis (Table 2, Step 15).

As presented Table 1A6, the population analysis will investigate if the literature is mainly populated by studies involving a limited population. This analysis will show the number of participants (NoP) of primary studies for three cases including participants with upper limb absence (ULA), able-bodied or asymptomatic participants (ABD) or both ULA and ABD (UNA). The distribution of references per study populations will be evidenced using case ratios defined as NoR involving participants with ULA per study involving ABD and UNA populations.

The population analysis will also investigate if the literature includes an important relative number of ABD participants. The balance between the participation of ABD and ULA population will be evidenced using sample ratio defined as the number of ABD participants per NoP with ULA in UNA population.

As detailed in Table 2C (Tasks 62–70), the cases and sample sizes will be extracted manually for each reference by reviewers considering information provided in the title and abstract and eventually the manuscript when needed. Preliminary observations showed that some quantitative studies may present excessively large sample sizes (i.e., phone surveys, queries of large databases) [35,36]. As a result, we cautiously recommend using the modified IQR method to identify possible outliers (e.g., cohort sizes above the upper bound [Third Quartile or Q3 + 40xIQR]). Such outliers may be excluded from statistical analyses to minimize potential biases introduced by unusually well-powered studies. The NoP per case will be represented using median and IRQ or mean and standard deviation depending on non-normal or normal distribution of the population, respectively.

#### Pertinence analysis (Table 2, Step 16).

As presented Table 1A7, the pertinence analysis will investigate if the literature does unevenly focus on care interventions and clinical outcomes. The analysis will show the distribution of references focusing on three care interventions (e.g., device, control, emerging solutions) and four clinical outcomes (e.g., neuromusculoskeletal pain, fitting satisfaction, ambidexterity level, functional ability).

As detailed in Table 2C (Tasks 71–76), associations between topics connected to care interventions and clinical outcomes will be identified automatically using keywords found in the titles and abstracts. References focusing on interventions and outcomes will be paired based on occurrence of keywords. An evidence score will combine the number of publications and level of evidence according to study design calculated for each association. Finally, evidence score for each association will be represented as a heatmap where the spectrum from light to dark will be proportional to the highest and lowest scores, respectively.

### Ethics

No ethical approval will be required for this subsequent review, as only information extracted from previously published studies will be considered.

## Discussion

### Innovative approach

Initially, we identified a series of alleged publication barriers commonly reported that could potentially limit the effectiveness of ULA rehabilitation programs. A recent publication suggests that confirming these barriers might involve examining an overwhelming large number of references. Conventional quantitative bibliometric or scientometric methods may not be well-equipped to handle the analysis of thousands of references. Hence, the need to design new metrics. For the first time, this review protocol outlined the literature macro-analyses required to characterize the alleged publication barriers in ULA rehabilitation.

### Protocol limitations

The limitations specific to each analysis are detailed in Table 1B. Mostly, the macro-analyses will overlook the position of authors within the byline, conflict of interest, the impact of the publications and the role of ABD population in study design and the contextual analysis of keywords. A key limitation that distinguishes literature macro-analyses from systematic reviews is lack of consideration with the methodological quality and rigor of studies (e.g., blinding, sample size, randomization statistical power, risks of bias) [37].

The characterization of the barriers will be limited by extrinsic barriers associated with the mining of the raw data extracted from various electronic sources. Accurately estimating the global population of individuals with ULA presents significant challenges. Consequently, the population datasets considered for the apportion analysis might lack accuracy. Inconsistent metadata across sources will require substantial efforts for the systematic extraction of names and affiliations, which might restrict the expertise and location analysis (e.g., important amounts of missing data).

The characterization of the barriers will also be limited by intrinsic factors associated with the calculations of the ratios and their interpretation. Several categorizations will rely on educated choices without sensitivity analyses (e.g., pyramids of expertise and evidence). At this stage, the proposed macro-analyses will not be stratified by level or cause of amputation. More importantly, interpretations of the outcomes will be inevitably subjective due to the lack of comparative data from other fields and the absence of established guidelines for conducting such macro-analyses [11,12].

### Dissemination plans

We foresee that the outcomes of future reviews implementing this protocol will be primarily published in scientific manuscripts, presented in international conferences and shared directly with stakeholders of ULA rehabilitation thought seminars or other scientific communication channels.

### Anticipated benefits

Future reviews implementing this protocol will be necessary to verify whether the suggested macro-analyses are practical and advantageous.

Nonetheless, we anticipated that findings from the literature macro-analyses focusing on ULA rehabilitation will provide insights into the underlying factors that impede the development of rigorous studies and clinical practice guidelines (e.g., scarcity of dedicated funding opportunities, difficulties to recruit large ULA populations). In the long term, repeating regular reviews based on these macro-analyses will be valuable for monitoring the evolution of barriers over time and assessing the impact of new global governance and policies.

This review protocol represents an initial step toward establishing a new framework for conducting literature macro-analyses within a given field. Future implementations of this protocol will provide benchmark ratios that may serve as references for subsequent comparative research. Hopefully, this will also encourage collegial discussions to set standards for assessing the developmental status of scientific literature across various disciplines.

Meanwhile, this protocol and subsequent reviews intended to offer valuable insights for stakeholders who shape the contemporary scientific ULA rehabilitation ecosystem. In practice, these efforts will support decision-makers in choosing effective strategies to advance bespoke rehabilitation initiatives and improve quality of life for individuals living with upper limb absence.

## Supporting information

S1 Table List of 344 keywords used to perform targeted searches (Step 3-Task 5). (PDF)

S2 Table Examples of focus, subtopic, source and syntax including keywords for (A) selected generic (Step 2-Task 4) and (B) targeted (Step 3-Task 6) searches considering population, intervention, comparator and outcome elements of studies (Step 2-Task 3) adapted from [3,20]. (PDF)

## Abbreviations

ABD: Population including able-bodied or asymptomatic participants
CINAHL: Cumulative Index of Nursing and Allied Health Literature
CSV: Comma Separated Values
ENL: EndNote 20 library (Clarivate Analysis LLC, United Kingdom)
IQR: Interquartile Range (Q1-Q4)
ISO: International Organization for Standardization
L: Level of evidence (L1-L7)
LMIC: Low- and middle-income countries
NoP: Number of participants
NoR/M: Number of references per million of individuals with ULA
NoR: Number of references
PICOS: Population, intervention, comparator, outcome and design elements of studies
PoA: Percentage of affiliations
PoR: Percentage of references
PRISMA: Preferred Reporting Items for Systematic Reviews and Meta-Analyses
PROSPERO: International prospective register of systematic reviews
RAF: Reference Acquisition Form
ULA: Population including individuals with upper limb absence
UNA: Population including both ULA and ABD cases
USA: United States of America
XLS: Excel spreadsheet (Microsoft, Microsoft 365, version 2507, USA)

## References

[1] Shahsavari H, Matourypour P, Ghiyasvandian S, Ghorbani A, Bakhshi F, Mahmoudi M, et al. Upper limb amputation; Care needs for reintegration to life: An integrative review. Int J Orthop Trauma Nurs. 2020;38:100773. doi: 10.1016/j.ijotn.2020.100773 32362398

[2] Resnik LJ, Borgia ML, Clark MA. Prevalence and predictors of unmet need for upper limb prostheses: An observational cohort study. J Prosthet Orthot. 2023;2023:10.1097/JPO.0000000000000477. doi: 10.1097/JPO.0000000000000477 37565066 PMC10411186

[3] Peters AA, Postema SG, Reneman MF, van der Sluis CK. Barriers and facilitators associated with musculoskeletal complaints in individuals with upper limb absence - focus group results and a scoping review. Disabil Rehabil. 2023;45(8):1352–62. doi: 10.1080/09638288.2022.2060335 35437071

[4] Dizon LML, Leochico CFD, Ramirez RS, Rey-Matias RR. A scoping review on upper extremity prostheses: Satisfaction, barriers, and their implications in resource-limited countries. Prosthet Orthot Int. 2025;49(1):105–10. doi: 10.1097/PXR.0000000000000347 38517392

[5] Yamamoto M, Chung KC, Sterbenz J, Shauver MJ, Tanaka H, Nakamura T, et al. Cross-sectional international multicenter study on quality of life and reasons for abandonment of upper limb prostheses. Plast Reconstr Surg Glob Open. 2019;7(5):e2205. doi: 10.1097/GOX.0000000000002205 31333938 PMC6571339

[6] Resnik L, Borgia M, Cancio J, Heckman J, Highsmith J, Levy C, et al. Upper limb prosthesis users: A longitudinal cohort study. Prosthet Orthot Int. 2021;45(5):384–92. doi: 10.1097/PXR.0000000000000034 34469939

[7] Smail LC, Neal C, Wilkins C, Packham TL. Comfort and function remain key factors in upper limb prosthetic abandonment: findings of a scoping review. Disabil Rehabil Assist Technol. 2021;16(8):821–30. doi: 10.1080/17483107.2020.1738567 32189537

[8] Gates DH, Engdahl SM, Davis A. Recommendations for the successful implementation of upper limb prosthetic technology. Hand Clin. 2021;37(3):457–66. 10.1016/j.hcl.2021.05.007 34253318

[9] Salminger S, Stino H, Pichler LH, Gstoettner C, Sturma A, Mayer JA, et al. Current rates of prosthetic usage in upper-limb amputees - have innovations had an impact on device acceptance?. Disabil Rehabil. 2022;44(14):3708–13. doi: 10.1080/09638288.2020.1866684 33377803

[10] Henderson E, Rehani M, Hebert JS. Sex and gender differences in upper extremity prosthesis rejection: A review of literature. Prosthet Orthot Int. 2024;48(3):300–14. doi: 10.1097/PXR.0000000000000330 38579197

[11] Liu Z, Wang M, Liu Q, Huang B, Teng Y, Li M, et al. Global trends and current status of amputation: Bibliometrics and visual analysis of publications from 1999 to 2021. Prosthet Orthot Int. 2024;48(5):603–15. doi: 10.1097/PXR.0000000000000271 37615606

[12] Saleib R-KM, Pekbay B, Verhofstad MHJ, Paping MA, Van Vledder MG, Van Waes OJF. Analyzing research trends and developments in osseointegration in patients with extremity amputations: Systematic bibliometric analysis and research recommendations. Prosthet Orthot Int. 2025;49(5):596–606. doi: 10.1097/PXR.0000000000000410 39514709 PMC12509445

[13] Yildiz KA, Shin AY, Kaufman KR. Interfaces with the peripheral nervous system for the control of a neuroprosthetic limb: a review. J Neuroeng Rehabil. 2020;17(1):43. 10.1186/s12984-020-00667-5 PMC7063740 32151268

[14] Donnelley CA, Shirley C, von Kaeppler EP, Hetherington A, Albright PD, Morshed S, et al. Cost analyses of prosthetic devices: A systematic review. Arch Phys Med Rehabil. 2021;102(7):1404-1415.e2. doi: 10.1016/j.apmr.2021.02.010 33711275

[15] Widehammar C, Lidström Holmqvist K, Hermansson L. Training for users of myoelectric multigrip hand prostheses: a scoping review. Prosthet Orthot Int. 2021;45(5):393–400. doi: 10.1097/PXR.0000000000000037 34456321

[16] Hashim NA, Abd Razak NA, Shanmuganathan T, Jaladin RA, Gholizadeh H, Abu Osman NA. On the use of virtual reality for individuals with upper limb loss: a systematic scoping review. Eur J Phys Rehabil Med. 2022;58(4):612–20. doi: 10.23736/S1973-9087.22.06794-6 35044131 PMC9987328

[17] Rydland J, Spiegel S, Wolfe O, Alterman B, Johnson JT, Wheaton LA. Neurorehabilitation in Adults With Traumatic Upper Extremity Amputation: A Scoping Review. Neurorehabil Neural Repair. 2022;36(3):208–16. doi: 10.1177/15459683211070277 34967259

[18] Sinke M, Chadwell A, Smit G. State of the art of prosthesis simulators for the upper limb: A narrative review. Ann Phys Rehabil Med. 2022;65(6):101635. doi: 10.1016/j.rehab.2022.101635 35091112

[19] Guémann M, Olié E, Raquin L, Courtet P, Risch N. Effect of mirror therapy in the treatment of phantom limb pain in amputees: A systematic review of randomized placebo-controlled trials does not find any evidence of efficacy. Eur J Pain. 2023;27(1):3–13. doi: 10.1002/ejp.2035 36094758 PMC10086832

[20] van der Sluis CK, Bongers RM. TIPS for scaling up research in upper limb prosthetics. Prosthesis. 2020;2(4):340–51. doi: 10.3390/prosthesis2040032

[21] Kelley MA, Benz H, Engdahl S, Bridges JFP. Identifying the benefits and risks of emerging integration methods for upper limb prosthetic devices in the United States: an environmental scan. Expert Rev Med Devices. 2019;16(7):631–41. doi: 10.1080/17434440.2019.1626231 31145868

[22] Sturma A, Stamm T, Hruby LA, Bischof B, Salminger S, Gstoettner C, et al. Rehabilitation of high upper limb amputees after Targeted Muscle Reinnervation. J Hand Ther. 2022;35(1):58–66. doi: 10.1016/j.jht.2020.10.002 33250398

[23] Prince M, Kenney LP, Howard D. A pin-array method for capturing tissue deformation under defined pressure distributions and its application to prosthetic socket design. Med Eng Phys. 2020;84:136–43. doi: 10.1016/j.medengphy.2020.08.003 32977910

[24] Shrestha S, Gautam S. Outcome of Three Dimensional Printed Functional Prostheses for Children with Upper Limb Deficiency in Nepal. Kathmandu Univ Med J (KUMJ). 2023;21(81):52–7. doi: 10.3126/kumj.v21i1.80524 37800426

[25] Heyns A, Jacobs S, Negrini S, Patrini M, Rauch A, Kiekens C. Systematic review of clinical practice guidelines for individuals with amputation: identification of best evidence for rehabilitation to develop the WHO’s package of interventions for rehabilitation. Arch Phys Med Rehabil. 2021;102(6):1191–7. doi: 10.1016/j.apmr.2020.11.019 33412108

[26] Negrello F, Stuart HS, Catalano MG. Hands in the Real World. Front Robot AI. 2019;6:147. 10.3389/frobt.2019.00147 PMC7806114 33501162

[27] Sensinger JW, Dosen S. A review of sensory feedback in upper-limb prostheses from the perspective of human motor control. Front Neurosci. 2020;14:345. doi: 10.3389/fnins.2020.00345 32655344 PMC7324654

[28] Nazari V, Zheng Y-P. Controlling upper limb prostheses using sonomyography (SMG): A review. Sensors (Basel). 2023;23(4):1885. doi: 10.3390/s23041885 36850483 PMC9959820

[29] Papaleo ED, D’Alonzo M, Fiori F, Piombino V, Falato E, Pilato F, et al. Integration of proprioception in upper limb prostheses through non-invasive strategies: A review. J Neuroeng Rehabil. 2023;20(1):118. doi: 10.1186/s12984-023-01242-4 37689701 PMC10493033

[30] ATScale Global Partnership for Assistive Technology. Product narrative: prostheses. 2020.

[31] McDonald CL, Westcott-McCoy S, Weaver MR, Haagsma J, Kartin D. Global prevalence of traumatic non-fatal limb amputation. Prosthet Orthot Int. 2021;45(2):105–14. doi: 10.1177/0309364620972258 33274665

[32] Caruso M, Harrington S. Prevalence of limb loss and limb difference in the United States: Implications for public policy. Coalition A, editor. USA: Amputee Coalition. 2024.

[33] Geil MD. JPO editor’s comments. J Prosthetics Orthotics. 2016;28(3):93.

[34] Beaudette R, Fiedler G. *Appropriateness of Sample Sizes in Published Research on Prosthetic Knee Componentry.* JPO. J Prosthetics Orthotics. 2018;30(2).

[35] Ro JS, et al. Trends in burden of work-related upper limb amputation in South Korea, 2004-2013: a nationwide retrospective cohort study. BMJ Open. 2019;9(11):e032793. 10.1136/bmjopen-2019-032793 PMC6886917 31748310

[36] Kurucan E, Thirukumaran C, Hammert WC. Trends in the management of traumatic upper extremity amputations. J Hand Surg Am. 2020;45(11):1086.e1-1086.e11. doi: 10.1016/j.jhsa.2020.05.006 32698980

[37] Boutron I, Page MJ, Higgins JP, Altman DG, Lundh A, Hróbjartsson A. Considering bias and conflicts of interest among the included studies. Cochrane Handbook for Systematic Reviews of Interventions. Wiley. 2019. 177–204. doi: 10.1002/9781119536604.ch7

