# Supplementary material for: Review of publication barriers in upper limb absence rehabilitation: A protocol for innovative literature macro-analyses
Source: PLoS One. 2026 Oct 23;21(10):e0360504. doi: 10.1371/journal.pone.0360504 (PMC13693909; doi:10.1371/journal.pone.0360504)
Supplement: Supplementary file 1 [file pone.0360504.s001.pdf]

Table S1. List of 344 keywords used to perform targeted searches (Step 3-Task 5).

1	*coding	173	middle*income
2	*comfort	174	military
3	*feedback	175	movement*
4	*intuitive control	176	MRI
5	*loop	177	myoelectric
6	*machine interface*	178	myoelectric* hand
7	*sensory	179	naive participant*
8	*tactile	180	naive subjects
9	1-handed	181	need*
10	2-handed	182	nerve interface*
11	3D print*	183	nerve* stimulation
12	3D print* socket	184	neuroprosthes*
13	3D*print*	185	neuroprostheses
14	9-test	186	neuroprosthetic*
15	abandon*	187	nonamputated limb pain
16	abandonment	188	non-amputee volunteers
17	able-bodied	189	non-dominant hand
18	acceptance	190	non-preferred hand
19	aching	191	non-prosthe* use*
20	active duty	192	number*amput*
21	actuation	193	occupational therapist
22	additive manufacturing	194	occurrence*amput*
23	adolescent	195	one-handed
24	adoption	196	osseointegrated*
25	affected limb pain	197	osseointegration
26	affordability	198	outcome* measure*
27	agonist-antagonist myoneural interface	199	paedia*
28	allotransplantation	200	pain relief
29	ambidexterity	201	paralympic games
30	ambidextrousness	202	patien* voice
31	amputated limb pain	203	patient-reported measur*
32	anchoring of prostheses into bone	204	pattern recognition
33	animal	205	performance*
34	armband*	206	performance-based outcome*
35	artificial intelligence	207	periphe* nerv*
36	auditory	208	physiotherapist
37	augmented reality	209	preference
38	avatar	210	preferred hand
39	bidirectional control	211	prescr*
40	bimanual	212	prevalence
41	bi-manual	213	printing
42	bimanual*	214	proprioception

43	bi-manual*	215	prosthe *use
44	bio*sens*	216	prosthe* control
45	bionic system*	217	prosthe* fit
46	bionic*	218	prosthe* fitting
47	bio-robotic devices	219	prosthe* handling
48	body*powered device*	220	prosthe* satisfaction
49	body*powered prosthe*	221	prosthe* usage
50	body-powered device*	222	prosthes* handling
51	body-powered prosthe*	223	prosthesis satisfaction
52	bone*anchor* limb*	224	prosthet* use
53	bone*anchor* prosth*	225	prosthetist
54	burden of disease	226	quality
55	care of military	227	quality of care
56	child*	228	quality of component*
57	classifier*	229	quality of hand*
58	clinical guidance	230	quality of harness*
59	clinical practice guideline*	231	quality of prosthe*
60	clinical setting	232	quality of socket
61	clinician	233	quality of suspension
62	co-creation	234	quality of terminal device*
63	co-design	235	quality*component*
64	cognitive attention	236	quality-oriented care
65	cognitive burden	237	questionnaire
66	cognitive effort	238	rabbit
67	computer-aided design	239	rate
68	congenital	240	rate*amput*
69	continuous attention	241	rating
70	contralateral hand	242	refuse * prosthe*
71	contralateral hand*	243	regene* nerve
72	contralateral limb pain	244	regenerative peripheral nerve interface*
73	contralateral pain	245	regional
74	co-occurring pain	246	rehabilitation cost*
75	cost*	247	rehabilitation expen*
76	cost-effectiveness	248	rejection*
77	cost-utility	249	residu* health
78	cybathlon	250	resource-limited healthcare
79	decision-making process	251	review of
80	deep learning	252	robotic
81	degree of freedom	253	ROM
82	demand*	254	rural
83	department of veterans affairs	255	safety
84	develop* countr*	256	satisfaction
85	dexter*	257	satisfaction with component*

86	dissatisfaction	258	satisfaction with harness*
87	dissatisfaction* hand	259	satisfaction with prosthe*
88	doctor	260	satisfaction with socket
89	dominant hand	261	satisfaction with suspension
90	effectiveness	262	satisfaction with terminal device*
91	electri* control	263	satisfaction* prosthe*
92	electrode*	264	satisfaction*hand*
93	electromyo*	265	self*report
94	electromyography	266	self-assessment*
95	EMG	267	semi-structured interview*
96	everyday action*	268	sensor*
97	everyday use	269	serious gam*
98	evoked sensation	270	service member*
99	exercise tolerance	271	shared decision-making
100	expectation*	272	sheep
101	expectations	273	shoulder limb pain
102	expenditure	274	shoulder pain
103	experience	275	simulator
104	experience with component*	276	simulator*
105	experience with harness*	277	single hand*
106	experience with prosthe*	278	sleeve
107	experience with socket	279	socioeconomic hardship
108	experience with suspension	280	socket
109	experience with terminal device*	281	socket design
110	experience* hand*	282	socket interface
111	experience*component*	283	socket score
112	experience*prosthe*	284	sono*
113	eye*tracking	285	sonomyo*
114	financial burden	286	soreness
115	fine manual skill*	287	sound arm pain
116	fine motor control	288	sound hand*
117	fine motor skill*	289	sound limb pain
118	functio* measur*	290	sound shoulder pain
119	function*	291	sport-specific prosthe*
120	function* test*	292	standard*
121	game*	293	standard* of care
122	game-based	294	stimulator*
123	gamification	295	surgeon
124	gaming	296	surgical cost*
125	gaz*	297	surgical expen*
126	general practioner	298	survey
127	guideline	299	system usability scale
128	guideline* for clinical practice	300	table-top prosthesis

129	hand dominance
130	hand transplantation
131	handling skill
132	haptic
133	harness
134	harness*
135	health economic*
136	health technology evaluat*
137	health*related quality of life
138	healthy hand
139	hobb*
140	HRQL
141	human*computer inter*
142	hybrid
143	improper socket fit
144	incidence*amput*
145	infant
146	in-laboratory
147	intact hand
148	intermanual transfer
149	interview*
150	labor* perform* metric*
151	laboratory test*
152	laterality
153	lateralization
154	learning phas*
155	life quality
156	limb pain
157	limb-intact participant
158	limb-intact subject
159	liner
160	LMIC
161	low back pain
162	lower*income
163	machine learning
164	magnet*
165	magnetic resonance image*
166	mastering control
167	mechanomyo*
168	mechatronic*
169	medical cost*
170	medical expen*
171	mental load
301	targeted muscle reinnervation
302	task
303	tasks
304	temperature
305	therapeutic modali*
306	therapists' opinions
307	three-dimensional prosthes*
308	three-dimensional-print*
309	toddler
310	touch
311	training
312	trauma*
313	treatment path*
314	ultrasound
315	unaffected hand
316	unaffected limb
317	unaffected limb pain
318	uniformity in treatment
319	unimanual
320	uni-manual
321	unrealistic experimental scenario*
322	upper limb prosthe*
323	upper limb* prosthe*
324	use of prosthe* hand
325	used of prosthe*
326	used of prosthe* hand
327	used of prosthes*
328	user experience*
329	user-centered approach
330	user-centred prosthe* research
331	user-computer inter*
332	user-driven prosthetic arm design
333	user's need
334	veteran*
335	video gam*
336	virtual control
337	virtual reality
338	virtualization
339	visual
340	visuomotor
341	war-wounded
342	wires*
343	workplace injur*

172	meta-synthes*	344	workshop
