# Supplementary material for: Review of publication barriers in upper limb absence rehabilitation: A protocol for innovative literature macro-analyses
Source: PLoS One. 2026 Oct 23;21(10):e0360504. doi: 10.1371/journal.pone.0360504 (PMC13693909; doi:10.1371/journal.pone.0360504)
Supplement: Supplementary file 2 [file pone.0360504.s002.pdf]

Table S1. Examples of focus, subtopic, source and syntax including keywords for (A) selected generic (Step 2-Task 4) and (B) targeted (Step 3-Task 6) searches considering population, intervention, comparator and outcome elements of studies (Step 2-Task 3) adapted from [3,20]

	Search, Focus, subtopic, source and syntax
**A-Examples of generic searches**	
**1**	**Population, Upper limb amputation rehabilitation, Google**<br>"upper limb amputation" and "rehabilitation"
**2**	**Population, Upper limb amputation rehabilitation, PubMed**<br>((((("Artificial Limbs" [Mesh] OR arm prosthe* [tiab] OR upper limb prosthe* [tiab] OR upper-limb prosthe* [tiab] OR upper extremity prosthe* [tiab] OR artificial arm* [tiab] OR hand prosthe* [tiab] OR artificial hand* [tiab])) AND (("Disability Evaluation" [Mesh] OR "International Classification of Functioning, Disability and Health" [Mesh] OR "Rehabilitation" [Mesh] OR control [tiab] OR "rehabilitation" [Subheading] OR "Treatment Outcome" [Mesh:NoExp] OR rehabilit* [tiab] OR function* [tiab] OR disabil* [tiab] OR outcome* [tiab] OR treatment* [tiab] OR training [tiab] OR activit* [tiab]))) NOT ((leg* [tiab] OR lower limb* [tiab] OR lower extremit* [tiab] OR foot [tiab] OR feet [tiab] OR finger*[tiab] OR tibia* [tiab] OR knee* [tiab] OR hip* [tiab] OR transfemoral [tiab] OR transtibial OR stroke [tiab] OR spinal cord [tiab] OR tetraplegia [tiab]))) AND (English[Language])
**3**	**Population, Upper limb amputation rehabilitation, PubMed**<br>(((arm prosthe* [tiab] OR upper limb* prosthe* [tiab] OR upper-limb prosthe* [tiab] OR upper extremity prosthe* [tiab] OR artificial arm [tiab] OR artificial arm [tiab] OR hand prosthe* [tiab] OR artificial hand* [tiab] OR upper limb ampu* [tiab] OR upper limb absence [tiab]) NOT (lower limb* [tiab] OR lower extremit* [tiab] OR thigh* [tiab] OR leg [tiab] OR foot [tiab] OR feet [tiab] OR toe [tiab] OR hip [tiab] OR femur [tiab] OR knee [tiab] OR tibia* [tiab] OR ankle* [tiab] OR femoral [tiab] OR transfemoral [tiab] OR transtibial [tiab] OR tibial [tiab] OR stroke [tiab] OR spinal cord [tiab] OR tetrapleg* [tiab]))) AND (English[Language])
**4**	**Outcome, Neuromusculoskeletal pain, PubMed**<br>(((("Artificial Limbs" [Mesh] OR arm prosthe* [tiab] OR upper limb prosthe* [tiab] OR upper-limb prosthe* [tiab] OR upper extremity prosthe* [tiab] OR artificial arm [tiab] OR artificial arms [tiab] OR hand prosthe* [tiab] OR artificial hand [tiab] OR artificial hands [tiab])) AND ("pain" [Mesh] OR phantom pain OR limb pain OR residu* pain OR contralateral pain OR shoulder pain OR neck pain OR back pain) NOT (leg [tiab] OR lower limb* [tiab] OR lower extremit* [tiab] OR foot [tiab] OR feet [tiab] OR finger*[tiab] OR tibia* [tiab] OR knee [tiab] OR hip [tiab] OR transfemoral [tiab] OR transtibial OR stroke [tiab] OR spinal cord [tiab] OR tetraplegia [tiab]))) AND (English[Language])
**5**	**Outcome, Fitting experience, PubMed**<br>(((("Artificial Limbs" [Mesh] OR arm prosthe* [tiab] OR upper limb prosthe* [tiab] OR upper-limb prosthe* [tiab] OR upper extremity prosthe* [tiab] OR artificial arm [tiab] OR artificial arms [tiab] OR hand prosthe* [tiab] OR artificial hand [tiab] OR artificial hands [tiab])) AND (prosthe* fitting[MeSH Terms] OR prosthe* fit[MeSH Terms] OR quality[tiab] OR satisfaction[tiab] OR adoption[tiab] OR rate[tiab] OR rating[tiab] OR experience[tiab] NOT quality of life[All Fields]) NOT (leg [tiab] OR lower limb* [tiab] OR lower extremit* [tiab] OR foot [tiab] OR feet [tiab] OR finger*[tiab] OR tibia* [tiab] OR knee [tiab] OR hip [tiab] OR transfemoral [tiab] OR transtibial OR stroke [tiab] OR spinal cord [tiab] OR tetraplegia [tiab]))) AND (English[Language])
**6**	**Outcome, Ambidexterity level, PubMed**

(((("Artificial Limbs" [Mesh] OR arm prosthe* [tiab] OR upper limb prosthe* [tiab] OR upper-limb prosthe* [tiab] OR upper extremity prosthe* [tiab] OR artificial arm [tiab] OR artificial arms [tiab] OR hand prosthe* [tiab] OR artificial hand [tiab] OR artificial hands [tiab])) AND ("handedness" [Mesh] OR contralateral hand OR contralateral hand OR one-handed OR intact arm OR uni-manual OR unimanual OR bi-manual OR bimanual OR single hand* [tiab]) NOT (leg [tiab] OR lower limb* [tiab] OR lower extremit* [tiab] OR foot [tiab] OR feet [tiab] OR finger*[tiab] OR tibia* [tiab] OR knee [tiab] OR hip [tiab] OR transfemoral [tiab] OR transtibial OR stroke [tiab] OR spinal cord [tiab] OR tetraplegia [tiab]))) AND (English[Language])

7 Outcome, Functional ability, PubMed

(((("Artificial Limbs" [Mesh] OR arm prosthe* [tiab] OR upper limb prosthe* [tiab] OR upper-limb prosthe* [tiab] OR upper extremity prosthe* [tiab] OR artificial arm [tiab] OR artificial arms [tiab] OR hand prosthe* [tiab] OR artificial hand [tiab] OR artificial hands [tiab])) AND ("handedness" [Mesh] OR contralateral hand OR contralateral hand OR one-handed OR intact arm OR uni-manual OR unimanual OR bi-manual OR bimanual OR single hand* [tiab]) NOT (leg [tiab] OR lower limb* [tiab] OR lower extremit* [tiab] OR foot [tiab] OR feet [tiab] OR finger*[tiab] OR tibia* [tiab] OR knee [tiab] OR hip [tiab] OR transfemoral [tiab] OR transtibial OR stroke [tiab] OR spinal cord [tiab] OR tetraplegia [tiab]))) AND (English[Language])

8 Intervention, Prosthetic care, PubMed

(((("Artificial Limbs" [Mesh] OR arm prosthe* [tiab] OR upper limb prosthe* [tiab] OR upper-limb prosthe* [tiab] OR upper extremity prosthe* [tiab] OR artificial arm [tiab] OR artificial arms [tiab] OR hand prosthe* [tiab] OR artificial hand [tiab] OR artificial hands [tiab])) AND (harness*[tiab] OR suspension[tiab] OR socket design[tiab] OR socket interface[tiab] OR 3D print* socket[tiab] OR prosthe* component*[tiab] OR passive prosthe*[tiab] OR body-powered device*[tiab] OR body-powered prosthe*[tiab] OR cable system[tiab] OR cable control mechanism*[tiab] OR electri* powered prosthe*[tiab] OR electri* controlled prosthe*[tiab] OR electri* control[tiab] OR electri* prosthes*[tiab] OR myoelectri* controlled prosthe*[tiab] OR myoelectri* device[tiab] OR controlled myoelectri*[tiab] OR microprocessor* controlled prosthe*[tiab] OR neuroprostheses[tiab] OR bionic system*[tiab] OR hybrid system*[tiab] OR hybrid prosthe*[tiab] OR activity*specific prosthe*[tiab] OR task*specific prosthe*[tiab] OR terminal device[tiab] OR bionic hand*[tiab] OR prosthetic hand[tiab] OR active wrist[tiab] OR hook terminal device[tiab] OR multi*articulat* hand[tiab] OR myoelectric* hand[tiab]) NOT (leg [tiab] OR lower limb* [tiab] OR lower extremit* [tiab] OR foot [tiab] OR feet [tiab] OR finger*[tiab] OR tibia* [tiab] OR knee [tiab] OR hip [tiab] OR transfemoral [tiab] OR transtibial OR stroke [tiab] OR spinal cord [tiab] OR tetraplegia [tiab]))) AND (English[Language])

B-Example of specific searches

9 Outcome, Neuromusculoskeletal pain, PubMed

((((arm prosthe* [tiab] OR upper limb* prosthe* [tiab] OR upper-limb prosthe* [tiab] OR upper extremity prosthe* [tiab] OR artificial arm [tiab] OR artificial arm [tiab] OR hand prosthe* [tiab] OR artificial hand* [tiab] OR upper limb ampu* [tiab] OR upper limb absence [tiab]) AND (residu* pain[MeSH Terms] OR phantom pain[MeSH Terms] OR affected limb pain[MeSH Terms] OR amputated limb pain[MeSH Terms] OR contralateral limb pain[MeSH Terms] OR shoulder limb pain[MeSH Terms] OR unaffected limb pain[MeSH Terms] OR sound limb pain[MeSH Terms] OR sound arm pain[MeSH Terms] OR sound shoulder pain[MeSH Terms] OR unaffected limb pain[MeSH Terms] OR sound limb pain[tiab] OR nonamputated limb pain[tiab] OR neck pain[tiab] OR back pain[tiab] OR low back pain[tiab] OR pain[tiab] OR

comfort[tiab] OR aching[tiab] OR soreness[tiab] OR pain relief[tiab] OR co-occurring pain[tiab]) NOT (digits [tiab] OR finger [tiab] OR lower limb* [tiab] OR lower extremit* [tiab] OR thigh* [tiab] OR leg [tiab] OR foot [tiab] OR feet [tiab] OR toe [tiab] OR hip [tiab] OR femur [tiab] OR knee [tiab] OR tibia* [tiab] OR ankle* [tiab] OR femoral [tiab] OR transfemoral [tiab] OR transtibial [tiab] OR tibial [tiab] OR stroke [tiab] OR spinal cord [tiab] OR tetrapleg* [tiab]))) AND (English[Language])

10 Outcome, Fitting experience, PubMed

(((arm prosthe* [tiab] OR upper limb* prosthe* [tiab] OR upper-limb prosthe* [tiab] OR upper extremity prosthe* [tiab] OR artificial arm [tiab] OR artificial arm [tiab] OR hand prosthe* [tiab] OR artificial hand* [tiab] OR upper limb ampu* [tiab] OR upper limb absence [tiab]) AND (quality of harness*[tiab] OR quality of suspension[tiab] OR quality of socket[tiab] OR experience with harness*[tiab] OR experience with suspension[tiab] OR experience with socket[tiab] OR satisfaction with harness*[tiab] OR satisfaction with suspension[tiab] OR satisfaction with socket[tiab] OR socket score[tiab] OR improper socket fit[tiab] OR quality of prosthe*[tiab] OR quality of component*[tiab] OR experience with prosthe*[tiab] OR experience with component*[tiab] OR satisfaction with prosthe*[tiab] OR satisfaction with component*[tiab] OR quality of terminal device*[tiab] OR experience with terminal device*[tiab] OR satisfaction with terminal device*[tiab] OR quality of hand*[tiab] OR experience* hand*[tiab] OR satisfaction hand*[tiab] OR dissatisfaction* hand[tiab] OR quality of prosthe*[tiab] OR quality*component*[tiab] OR experience prosthe*[tiab] OR experience component*[tiab] OR satisfaction* prosthe*[tiab] OR satisfaction with component*[tiab] OR prosthe* satisfaction[tiab] OR system usability scale[tiab]) NOT (digits [tiab] OR finger* [tiab] OR lower limb* [tiab] OR lower extremit* [tiab] OR thigh* [tiab] OR leg [tiab] OR foot [tiab] OR feet [tiab] OR toe [tiab] OR hip [tiab] OR femur [tiab] OR knee [tiab] OR tibia* [tiab] OR ankle* [tiab] OR femoral [tiab] OR transfemoral [tiab] OR transtibial [tiab] OR tibial [tiab] OR stroke [tiab] OR spinal cord [tiab] OR tetrapleg* [tiab]))) AND (English[Language])

11 Outcome, Ambidexterity level, PubMed

(((arm prosthe* [tiab] OR upper limb* prosthe* [tiab] OR upper-limb prosthe* [tiab] OR upper extremity prosthe* [tiab] OR artificial arm [tiab] OR artificial arm [tiab] OR hand prosthe* [tiab] OR artificial hand* [tiab] OR upper limb ampu* [tiab] OR upper limb absence [tiab]) AND (prosthe* handling[tiab] OR used of prosthe*[tiab] OR prosthe *use[tiab] OR used of prosthe* hand[tiab] OR contralateral hand*[tiab] OR sound hand*[tiab] OR unaffected hand[tiab] OR intact hand[tiab] OR healthy hand[tiab] OR bi-manual*[tiab] OR bimanual*[tiab] OR 2-handed[tiab] OR handedness[tiab] OR laterality[tiab] OR ambidexterity[tiab] OR ambidextrousness[tiab] NOT preferred hand[tiab] NOT non-preferred hand[tiab] NOT dominant hand[tiab] NOT hand dominance[tiab] NOT non-dominant hand[tiab] NOT lateralization[tiab] NOT intermanual transfer[tiab] NOT 1-handed[tiab]) NOT (digits [tiab] OR finger* [tiab] OR lower limb* [tiab] OR lower extremit* [tiab] OR thigh* [tiab] OR leg [tiab] OR foot [tiab] OR feet [tiab] OR toe [tiab] OR hip [tiab] OR femur [tiab] OR knee [tiab] OR tibia* [tiab] OR ankle* [tiab] OR femoral [tiab] OR transfemoral [tiab] OR transtibial [tiab] OR tibial [tiab] OR stroke [tiab] OR spinal cord [tiab] OR tetrapleg* [tiab]))) AND (English[Language])

12 Outcome, Functional ability, PubMed

(((arm prosthe* [tiab] OR upper limb* prosthe* [tiab] OR upper-limb prosthe* [tiab] OR upper extremity prosthe* [tiab] OR artificial arm [tiab] OR artificial arm [tiab] OR hand prosthe* [tiab] OR artificial hand* [tiab] OR upper limb ampu* [tiab] OR upper limb absence [tiab]) AND (dexter*[tiab] OR fine motor skill*[tiab] OR fine manual skill*[tiab] OR fine motor control[tiab] OR handling skill[tiab] OR function[tiab] OR dexterity[tiab]

OR dexterous[tiab] OR daily task*[tiab] OR daily activit*[tiab] OR activit* of daily[tiab] OR range of movement*[tiab] OR gross motor skill*[tiab] OR gross manual skill*[tiab] OR everyday task*[tiab] OR daily task*[tiab] OR daily activit*[tiab] OR activit* of daily[tiab] OR everyday task*[tiab] OR everyday action*[tiab] OR everyday use[tiab] OR performance*[tiab] OR movement*[tiab] OR function*[tiab] OR task*[tiab]) NOT (digits [tiab] OR finger* [tiab] OR lower limb* [tiab] OR lower extremit* [tiab] OR thigh* [tiab] OR leg [tiab] OR foot [tiab] OR feet [tiab] OR toe [tiab] OR hip [tiab] OR femur [tiab] OR knee [tiab] OR tibia* [tiab] OR ankle* [tiab] OR femoral [tiab] OR transfemoral [tiab] OR transtibial [tiab] OR tibial [tiab] OR stroke [tiab] OR spinal cord [tiab] OR tetrapleg* [tiab]))) AND (English[Language])

13 Intervention, Prosthetic device, PubMed

(((arm prosthe* [tiab] OR upper limb* prosthe* [tiab] OR upper-limb prosthe* [tiab] OR upper extremity prosthe* [tiab] OR artificial arm [tiab] OR artificial arm [tiab] OR hand prosthe* [tiab] OR artificial hand* [tiab] OR upper limb ampu* [tiab] OR upper limb absence [tiab]) AND (socket[tiab] OR suspension[tiab] OR harness[tiab] OR sleeve[tiab] OR liner[tiab] OR passive prosthe*[tiab] OR passive device[tiab] OR active wrist[tiab] OR hook terminal device[tiab] OR multi*articulat* hand[tiab] OR passive prosthe*[tiab] OR passive device[tiab] OR body*powered device*[tiab] OR body*powered prosthe*[tiab] OR cable system[tiab] OR cable control mechanism*[tiab] OR electri* powered prosthe*[tiab] OR electri* controlled prosthe*[tiab] OR electri* prosthes*[tiab] OR myoelectri* controlled prosthe*[tiab] OR myoelectri* device[tiab] OR controlled myoelectri*[tiab] OR microprocessor* controlled prosthe*[tiab] OR neuroprosthes*[tiab] OR hybrid[tiab] OR hybrid system*[tiab] OR hybrid prosthe*[tiab] OR activity* specific prosthe*[tiab] OR task* specific prosthe*[tiab] OR hobb*[tiab] OR cybathlon[tiab] OR paralympic games[tiab] OR sport-specific prosthe*[tiab] OR terminal device[tiab] OR prosthetic hand[tiab] OR prosthe* component*[tiab] OR bionic hand*[tiab] OR degree of freedom[tiab]) NOT (digits [tiab] OR finger* [tiab] OR lower limb* [tiab] OR lower extremit* [tiab] OR thigh* [tiab] OR leg [tiab] OR foot [tiab] OR feet [tiab] OR toe [tiab] OR hip [tiab] OR femur [tiab] OR knee [tiab] OR tibia* [tiab] OR ankle* [tiab] OR femoral [tiab] OR transfemoral [tiab] OR transtibial [tiab] OR tibial [tiab] OR stroke [tiab] OR spinal cord [tiab] OR tetrapleg* [tiab]))) AND (English[Language])

14 Intervention, Prosthetic control, PubMed

(((arm prosthe* [tiab] OR upper limb* prosthe* [tiab] OR upper-limb prosthe* [tiab] OR upper extremity prosthe* [tiab] OR artificial arm [tiab] OR artificial arm [tiab] OR hand prosthe* [tiab] OR artificial hand* [tiab] OR upper limb ampu* [tiab] OR upper limb absence [tiab]) AND (sensor*[tiab] OR electrode*[tiab] OR wires*[tiab] OR bio*sens*[tiab] OR sonomyo*[tiab] OR mechanomyo*[tiab] OR electromyo*[tiab] OR magnet*[tiab] OR stimulator*[tiab] OR armband*[tiab] OR prosthe* control[tiab] OR *loop[tiab] OR eye*tracking[tiab] OR gaz*[tiab] OR haptic[tiab] OR temperature[tiab] OR *feedback[tiab] OR proprioception[tiab] OR visuomotor[tiab] OR *tactile[tiab] OR auditory[tiab] OR visual[tiab] OR *sensory[tiab] OR touch[tiab] OR *intuitive control[tiab] OR bidirectional control[tiab] OR evoked sensation[tiab] OR electromyography[tiab] OR deep learning[tiab] OR machine learning[tiab] OR actuation[tiab] OR EMG[tiab] OR pattern recognition[tiab] OR myoelectric[tiab] OR *coding[tiab] OR classifier*[tiab] OR artificial intelligence[tiab] OR simulator*[tiab] OR table-top prosthesis[tiab]) NOT (digits [tiab] OR finger* [tiab] OR lower limb* [tiab] OR lower extremit* [tiab] OR thigh* [tiab] OR leg [tiab] OR foot [tiab] OR feet [tiab] OR toe [tiab] OR hip [tiab] OR femur [tiab] OR knee [tiab] OR tibia* [tiab] OR ankle* [tiab] OR femoral [tiab] OR transfemoral [tiab] OR transtibial [tiab] OR tibial [tiab] OR stroke [tiab] OR spinal cord [tiab] OR tetrapleg* [tiab]))) AND (English[Language])
